# Comparing Self Monitoring Blood Glucose Devices and Laboratory Tests: Over 25 Years Experience

**DOI:** 10.7759/cureus.6268

**Published:** 2019-12-01

**Authors:** Yuko Harada, Keiichi Harada, Paul Chin

**Affiliations:** 1 Internal Medicine, Harada Naika Clinic, Kawasaki, JPN

**Keywords:** smbg, capillary blood

## Abstract

Self-monitoring blood glucose (SMBG) devices have been widely used in medical practice for decades. However, there are certain gaps between SMBG device readings and venous blood glucose levels. Here, 3,532 measurement data points were recorded over 25 years to compare SMBG device readings and venous blood glucose levels. The mean absolute difference (MAD) or the GAP was 10.9 mg/dL, and the mean absolute relative difference (MARD) was 8.3%. The absolute relative difference (ARD) (%) and absolute difference (AD or GAP) (mg/dL) coefficients of variation (CV) (%) of 100% indicate high variance between the capillary BG and venous true BG data. There was a slight skewing of MAD and MARD to the lower body mass index (BMI) side, the higher age side, and the female gender side. There were 41 data points that showed unacceptable gaps of over 50 mg/dL. Such large differences may cause incorrect medications or treatments. All healthcare providers should be aware of the gap between SMBG device readings and venous blood glucose levels.

## Introduction

Self-monitoring blood glucose (SMBG) has been recommended to be routinely used for successful diabetes management and therapy in international guidelines [1-2]. The use of electrode systems to measure blood glucose was advocated by Clark and Lyons in 1962, and the first glucometer (SMBG device) was manufactured in 1986 which was introduced in Japan in 1991 [3-4]. It is known that there are certain gaps between the readings of glucometer and actual blood glucose levels. However, the prompt result of glucometer is more convenient than laboratory testing, which requires a long time. Thus, SMBG devices have been widely used in medical practice, from patients’ homes to hospital emergency rooms.

In medical office practice, laboratory test results usually take several days because the samples are sent to laboratory agencies. The use of glucometer is the only way to immediately measure the patients’ glucose level in medical offices. Over 25 years ago in a private medical clinic, a noted hematologist started to measure blood glucose level simultaneously by both SMBG device and venous blood test, because he believed the capillary blood glucose (local blood sugar) measured by SMBG device should be different from the venous glucose level (systemic blood sugar).

Here we report the extensive analyses of the data from a retrospective study comparing glucometer and laboratory tests. This is the largest and the most extensive database and analyses comparing capillary blood SMBG device measurements and venous blood glucose laboratory testing.

## Materials and methods

From 1993 through 2019, 5,300 patients visited this general internal medicine clinic. Since the doctors were not endocrinologists, many of the patients were non-diabetic. All of the diabetic patients had type 2 diabetes, and there were several insulin users.

3,532 measurement data points for blood glucose (BG) were recorded and compiled from 81 diabetes patients over a period of over 25 years. In order to minimize human error in measuring blood sugar, trained nurses and doctors performed the capillary finger pricks and blood analyses with self-monitoring blood glucose (SMBG) device, and immediately drew venous blood from the patients’ forearms. The nurses and doctors carefully followed the instruction manuals for each device utilized. All the fingers and forearms were disinfected with alcohol before taking samples. The venous blood samples were immediately taken to major blood test laboratories in order to measure blood glucose levels (True BG) and hemoglobin A1C (HbA1c). During the 25 years, five SMBG devices were utilized: one SMBG device for each of three brands, and two generations of SMBG devices for the fourth brand (Device M).

These data were parameterized according to capillary BG vs. True BG, AD (GAP) (mg/dL) distribution, capillary BG vs. True BG (mg/dL) AD (GAP) - 50 mg/dL and over, glucometer, body mass Index (BMI) (kg/m^2^), age, and gender.

Absolute relative difference (ARD) (%), mean ARD (MARD) (%), ARD coefficient of variation (CV) (%), and absolute difference (AD) (the GAP) (mg/dL), mean AD (MAD) (mg/dL), and AD coefficient of variation (CV) (%) were calculated.

ARD (%) is the absolute value of the difference between SMBG device measurements (capillary BG) and True BG divided by True BG. MARD (%) is the mean of the ARDs. ARD coefficient of variation (CV) (%) is the standard deviation of the ARDs divided by the average of the ARDs. AD (mg/dL) is the absolute value of the difference between capillary BG and True BG. MAD (mg/dL) is the mean of the ADs. AD coefficient of variation (CV) (%) is the standard deviation of the ADs divided by the average of the ADs.

Data tables and their corresponding charts are displayed below.

## Results

Capillary blood glucose vs. true blood glucose distribution

**Figure 1:**
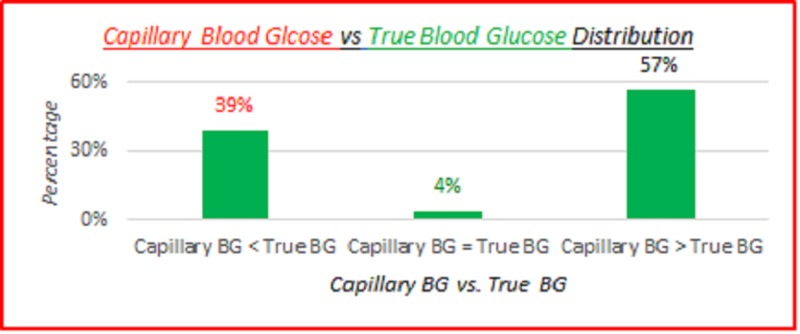
Capillary blood glucose vs. true blood glucose distribution BG: blood glucose

The data measurements were recorded in each patient's chart. Then the data were aggregated and analyzed retrospectively. The data shows SMBG device measurements underestimated true blood glucose (BG) 39% of the time, matched 4% of the time and over-estimated true BG 57% of the time. This distribution is displayed in the data table and chart shown below in Table 1 and Figure 1.

**Table 1:** Capillary blood glucose vs. true blood glucose distribution BG: blood glucose

Observations: 3,532 data points				
Capillary BG < True BG				39%
Capillary BG = True BG				4%
Capillary BG > True BG				57%

Absolute difference (AD or GAP) (mg/dL) distribution

**Figure 2:**
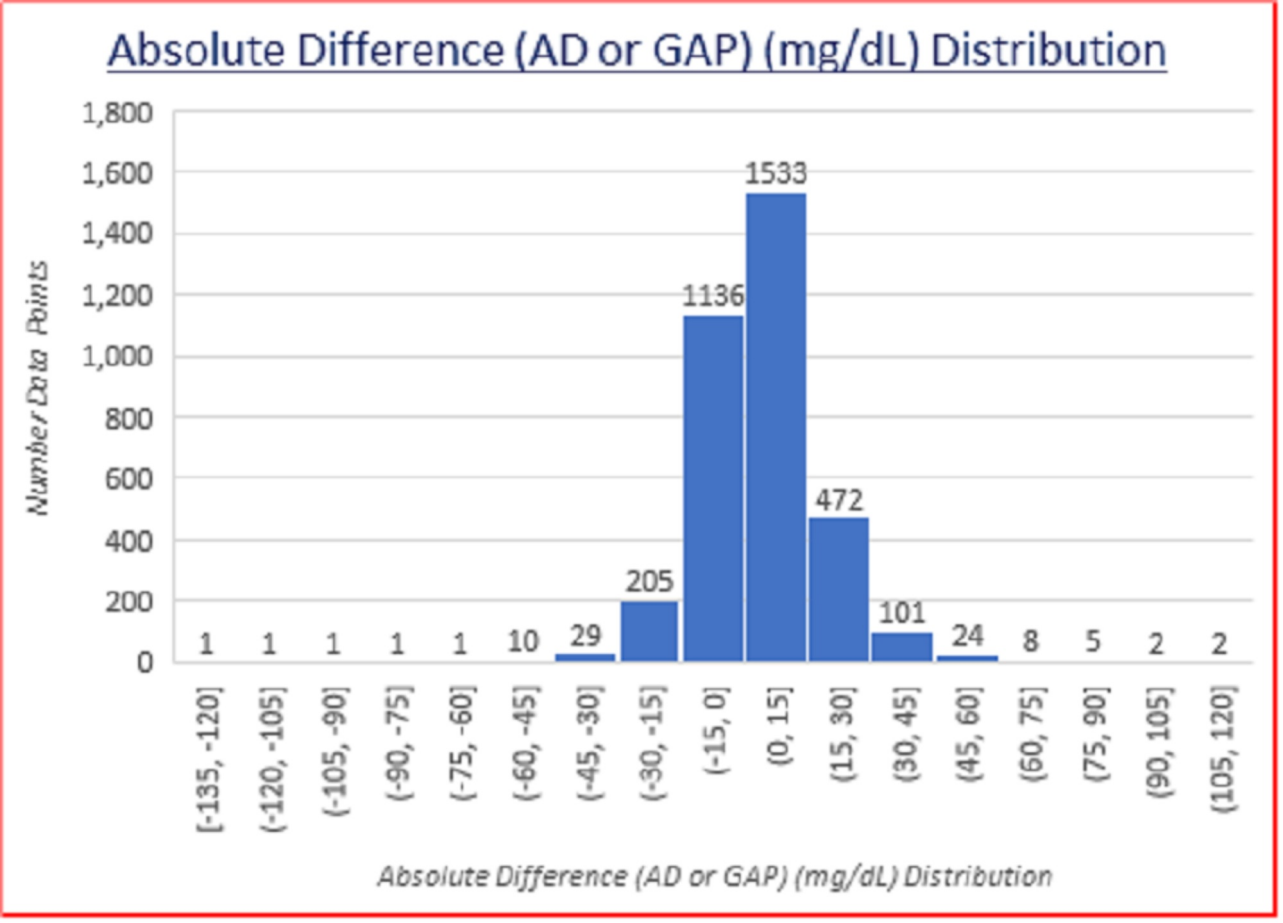
Absolute difference (AD or GAP) (mg/dL) distribution

The data distribution of the absolute difference (AD or GAP) (mg/dL) is shown below in Figure 2.

Capillary BG vs. true BG (mg/dL) absolute difference (AD or GAP) of unacceptable GAP of 50 mg/dL and over

**Figure 3:**
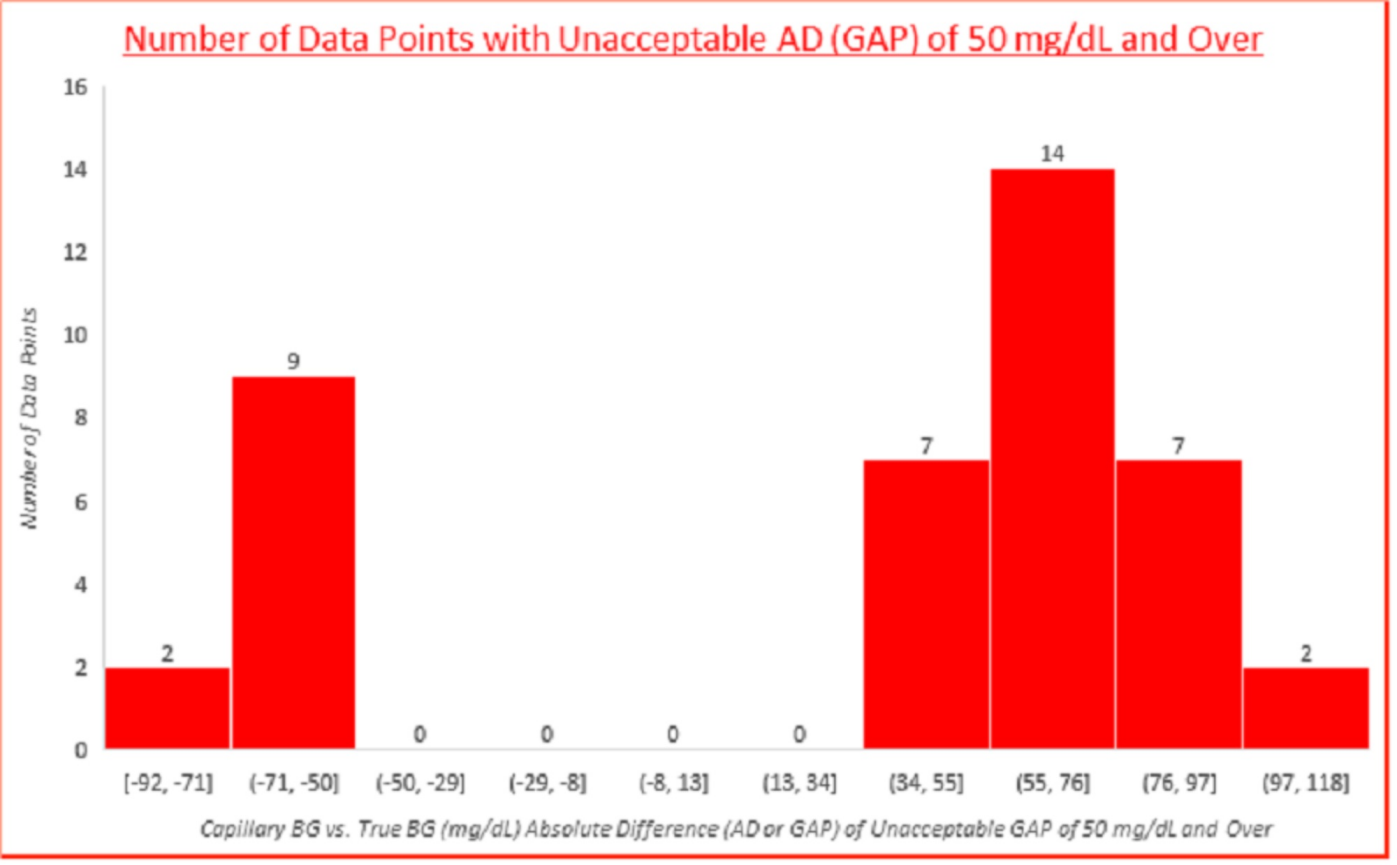
Capillary BG vs. true BG (mg/dL) absolute difference (AD or GAP) of unacceptable GAP of 50 mg/dL and over BG: blood glucose

The data distribution of the capillary BG vs. true BG (mg/dL) absolute difference (AD or GAP) of unacceptable GAP of 50 mg/dL and over is shown below in Figure 3. These could result in incorrect medication. The largest GAP was a triple-digit 118 mg/dL.

Absolute relative difference (ARD) (%) and absolute difference (AD or GAP) (mg/dL) coefficients of variation (CV) (%)

Absolute relative difference (ARD) (%) and absolute difference (AD or GAP) (mg/dL) coefficients of variation (%) of 100% indicate high variance between the capillary BG and venous true BG data. The coefficient of variation is defined as the standard deviation divided by the mean. These are displayed in the data table shown below in Table 2.

**Table 2:** Absolute relative difference (%) and absolute difference (AD or GAP) (mg/dL) coefficients of variation (%) SMBG: self-monitoring blood glucose; ARD: absolute relative difference; MARD: mean ARD; AD: absolute difference; MAD: mean absolute difference; CV: coefficient of variation

# Data pts	SMBG Device	MARD (%)	ARD CV (%)	MAD (mg/dL)	AD CV (%)	
3,532	All	8.3%	104%	10.9	102%	high variance

SMBG devices (glucometer)

**Figure 4:**
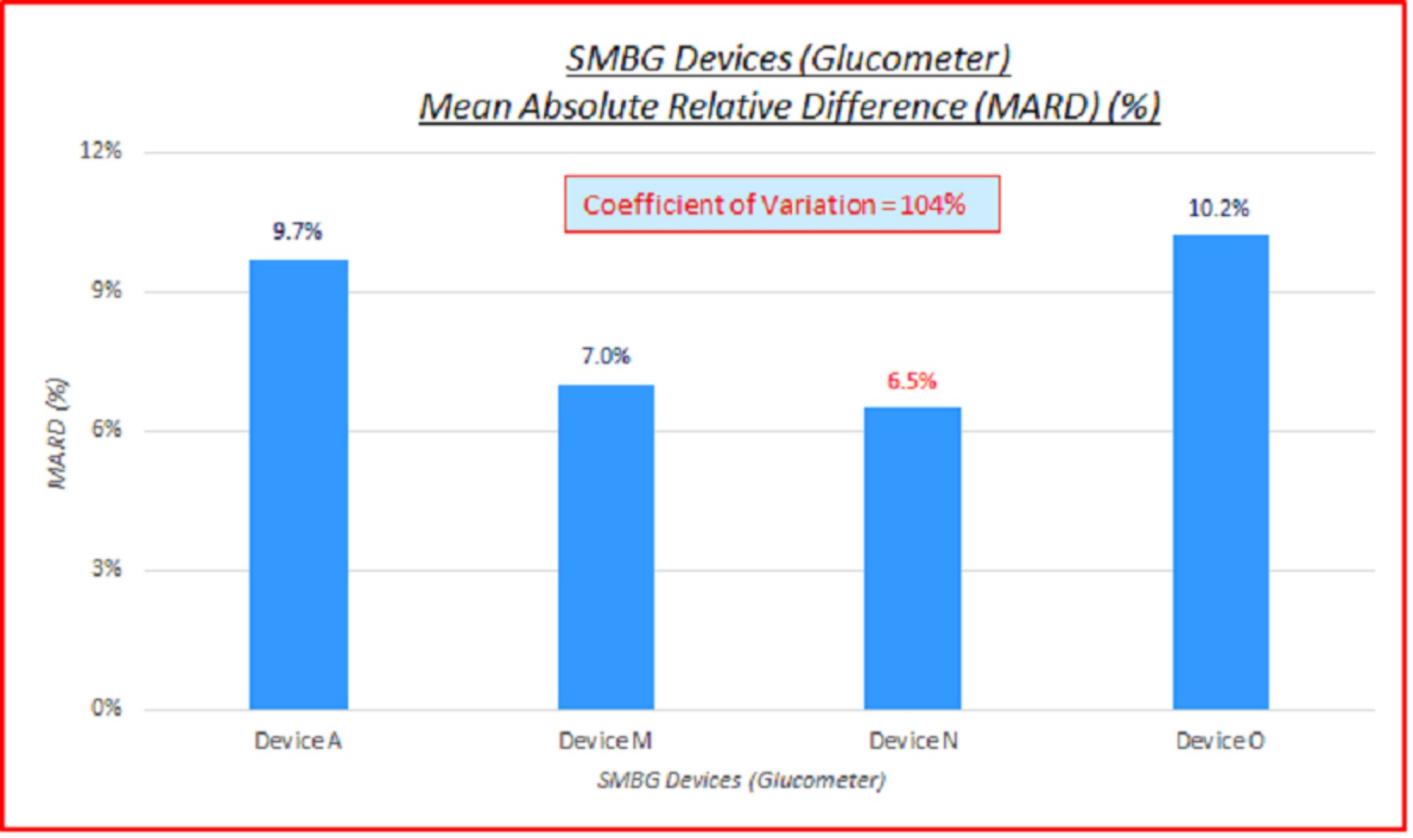
SMBG devices (glucometer) MARD (%) SMBG: self-monitoring blood glucose, MARD: mean absolute relative difference

**Figure 5:**
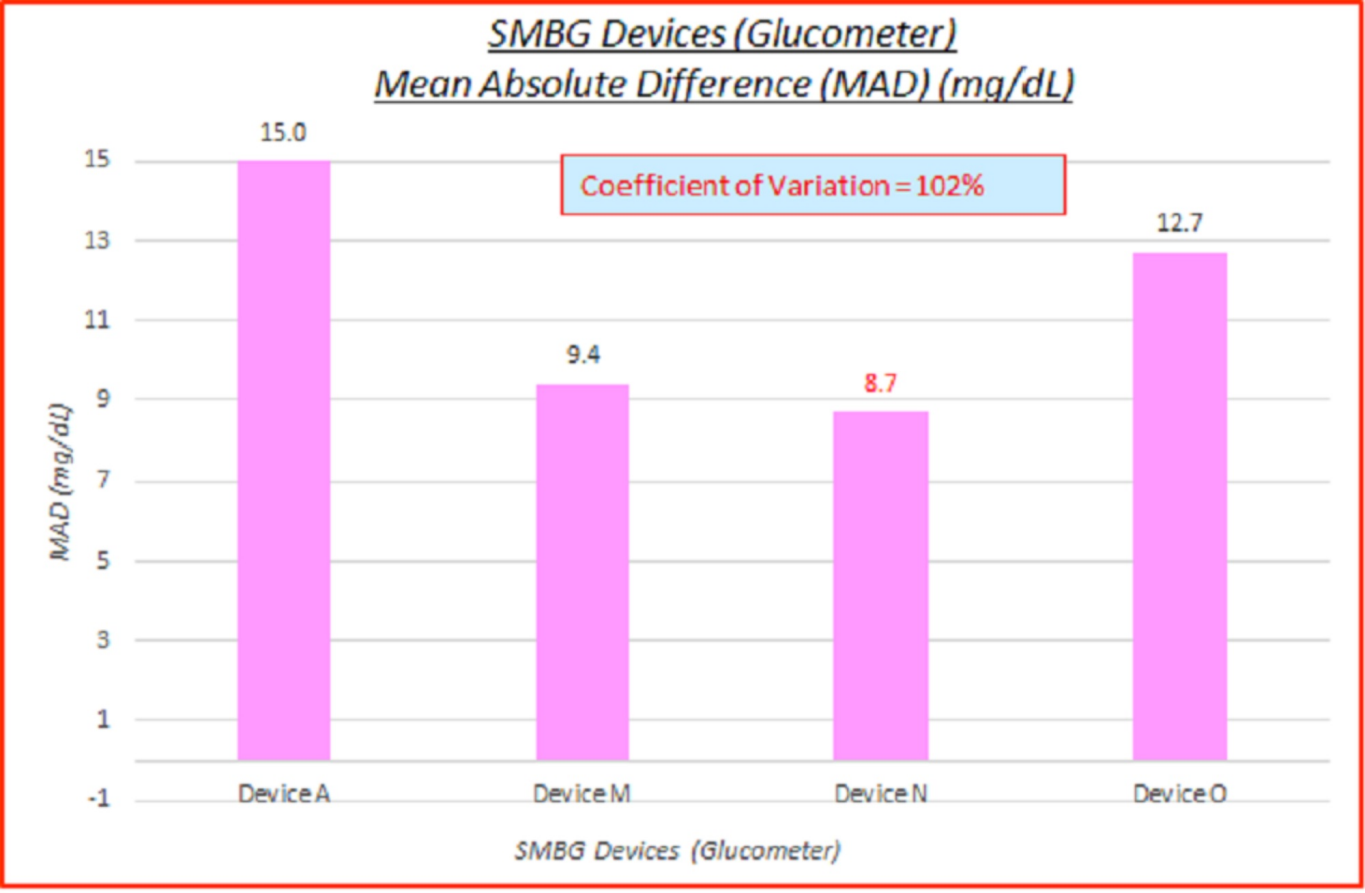
SMBG devices (glucometer) mean absolute difference (MAD) (%) SMBG: self-monitoring blood glucose; MAD: mean absolute difference

Five SMBG devices were utilized: one SMBG device for each of three brands, and two generations of SMBG devices for the fourth brand (Device M). Data parameterized according to SMBG devices (glucometer) are fairly consistent. ARD (%) and absolute difference (AD or GAP) (mg/dL) coefficients of variation (%) of 100% indicate high variance between the capillary BG and venous true BG data. These are displayed in the data table and charts below in Table 3 and Figures 4-5.

**Table 3:** SMBG devices (glucometer), absolute relative difference (ARD) (%) and absolute difference (AD or GAP) (mg/dL) coefficients of variation (%) SMBG: self-monitoring blood glucose; ARD: absolute relative difference; MARD: mean absolute relative difference; AD: absolute difference; MAD: mean absolute difference; CV: coefficient of variation

# Data pts	SMBG Device	MARD (%)	ARD CV (%)	MAD (mg/dL)	AD CV (%)
3,532	All	8.3%	104%	10.9	102%
244	Device A	9.7%	113%	15.0	101%
1,257	Device M	7.0%	97%	9.4	94%
687	Device N	6.5%	85%	8.7	105%
1,344	Device O	10.2%	102%	12.7	98%

Body mass index (BMI)

**Figure 6:**
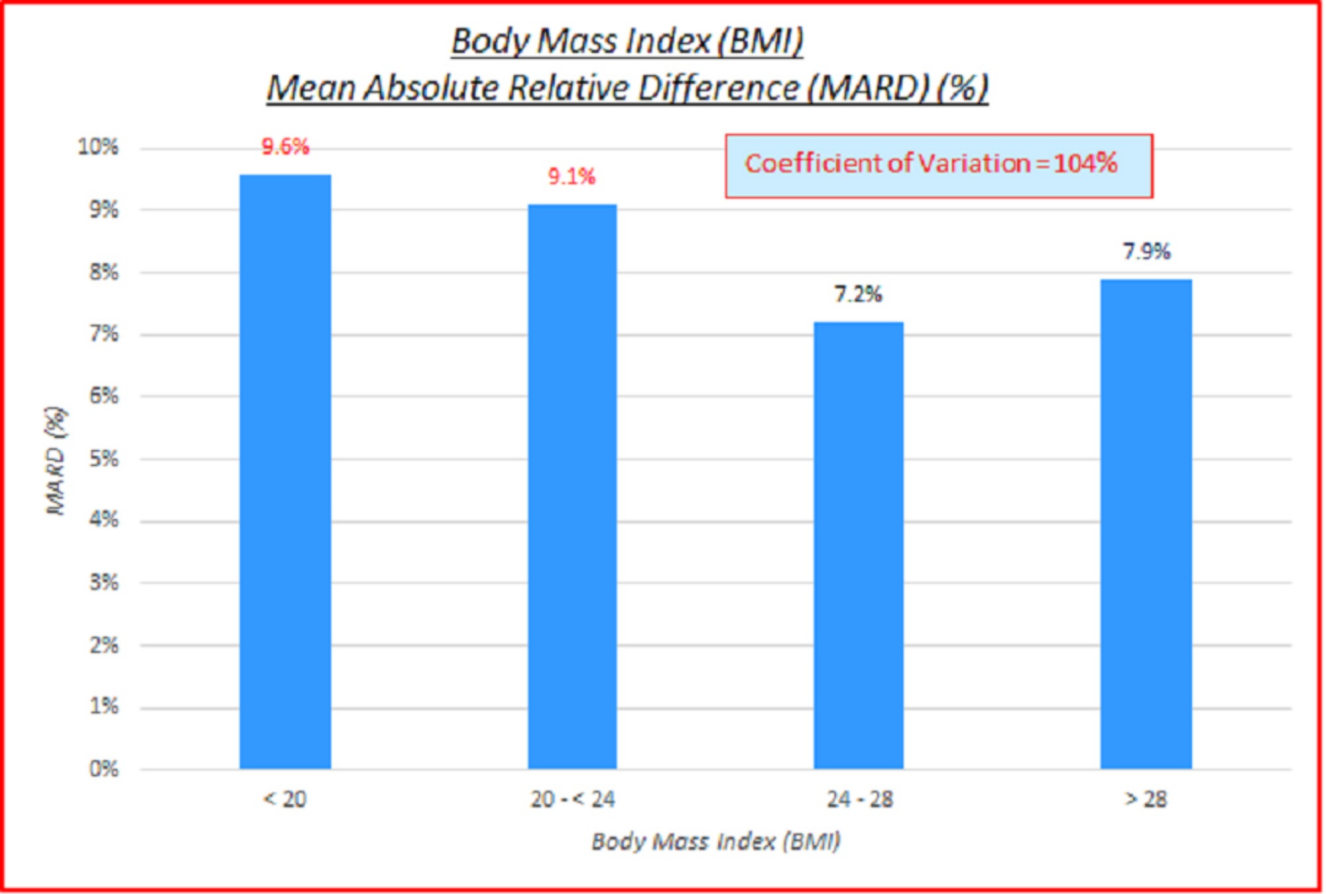
Body mass index (BMI) mean absolute relative difference (MARD) (%)

**Figure 7:**
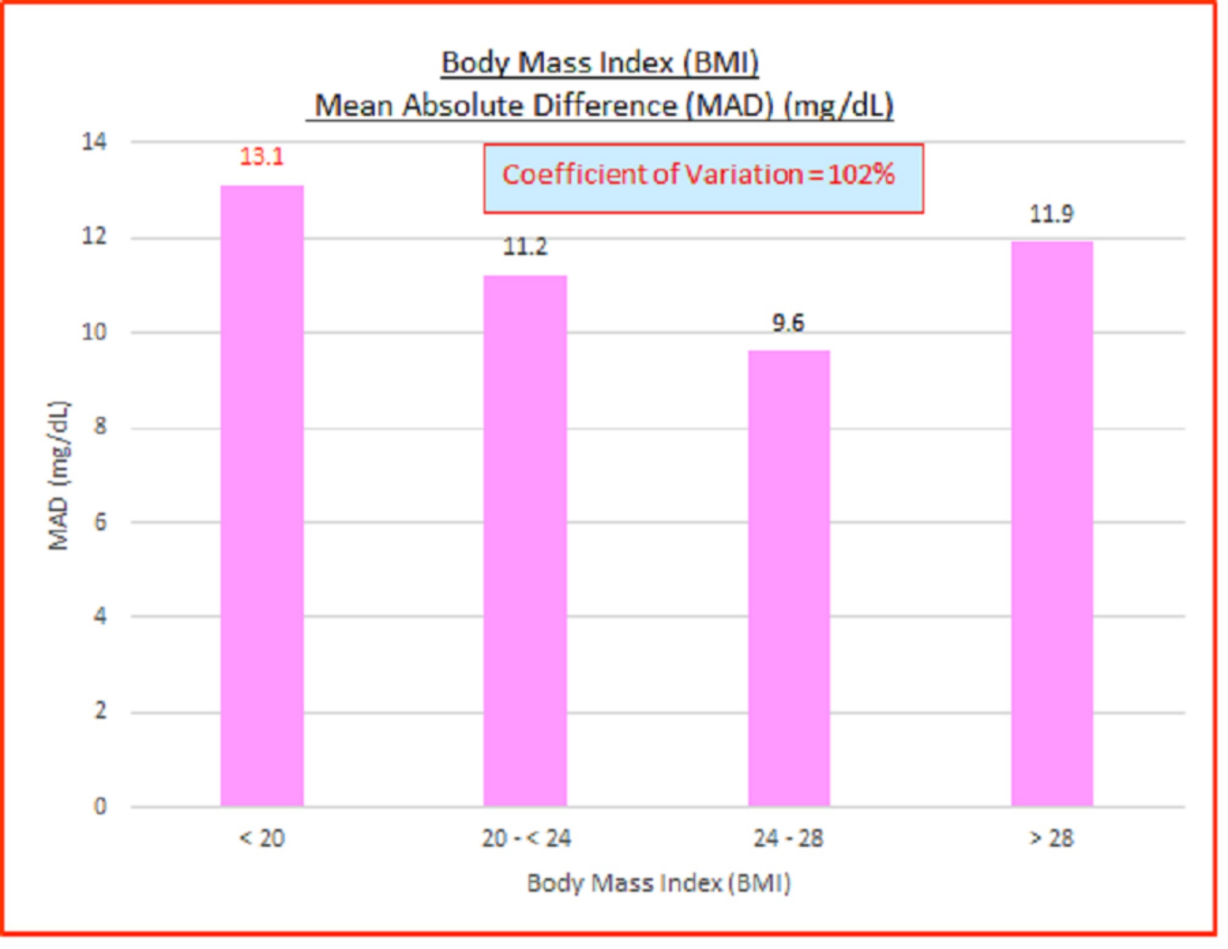
Body mass index (BMI) mean absolute difference (MAD)

Data parameterized according to BMI (kg/m^2^) are fairly consistent. ARD (%) and absolute difference (AD or GAP) (mg/dL) coefficients of variation (%) of 100% indicate high variance between the capillary BG and venous true BG data. They show a slight skewing of mean absolute relative difference (MARD) and mean absolute difference (MAD) to the lower BMI side. These are displayed in the data table and charts below in Table 4 and Figures 6-7.

**Table 4:** Body mass index (BMI), absolute relative difference (ARD) (%), absolute difference (AD or GAP) (mg/dL), coefficients of variation (%) ARD: absolute relative difference; MARD: mean absolute relative difference; AD: absolute difference; MAD: mean absolute difference, CV: coefficient of variation

# Data points	BMI (kg/m^2^)	MARD (%)	ARD CV (%)	MAD (mg/dL)	AD CV (%)
3,532	All	8.3%	104%	10.9	102%
485	< 20	9.6%	100%	13.1	95%
1,425	20 - < 24	9.1%	111%	11.2	101%
1,261	24 - 28	7.2%	92%	9.6	104%
325	> 28	7.9%	96%	11.9	98%
36	N/A				

Age

**Figure 8:**
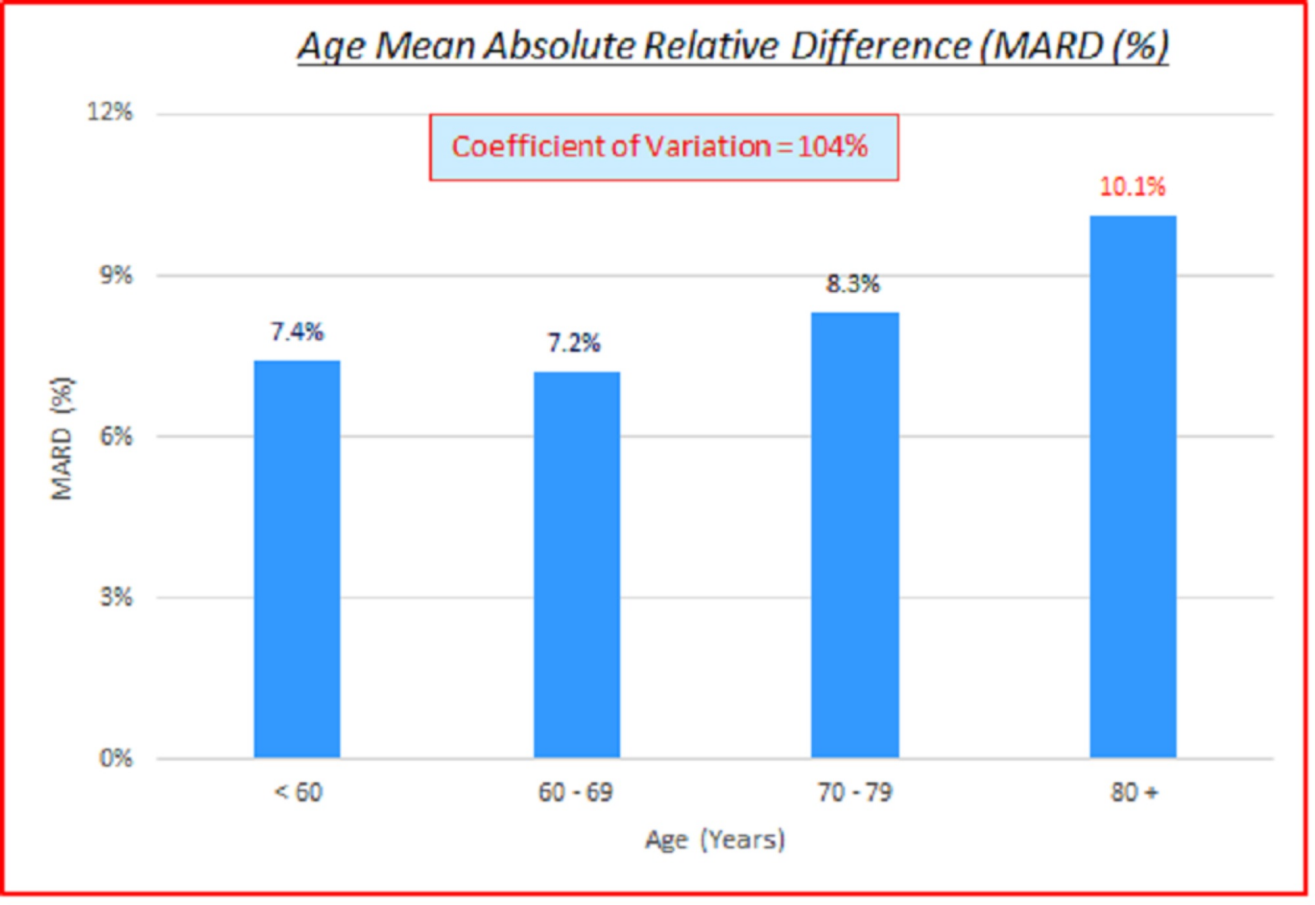
Age MARD (%) MARD: mean absolute relative difference

**Figure 9:**
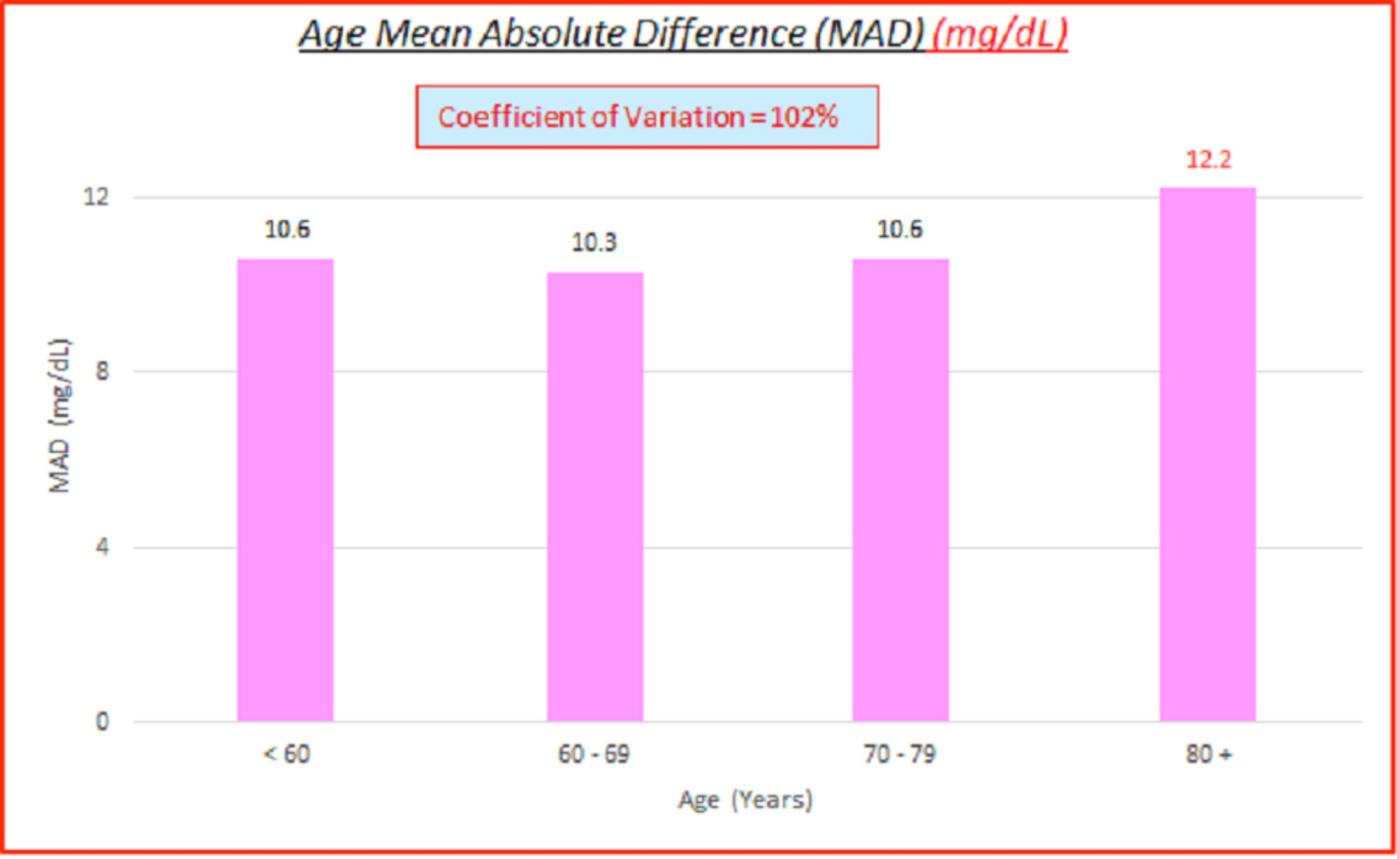
Age MAD MAD: mean absolute difference

Data parameterized according to age are fairly consistent. ARD (%) and absolute difference (AD or GAP) (mg/dL) Coefficients of variation (%) of 100% indicate high variance between the capillary BG and venous true BG data. They show a slight skewing of MARD and mean MAD to the higher age side. These are displayed in the data table and charts shown below in Table 5 and Figures 8-9.

**Table 5:** Age absolute relative difference (%) and absolute difference (AD or GAP) (mg/dL) coefficients of variation (%) ARD: absolute relative difference; MARD: mean ARD; AD: absolute difference; MAD: mean AD; CV: coefficient of variation

# Data pts	Age	MARD (%)	ARD CV (%)	MAD (mg/dL)	AD CV (%)
3,532	All	8.3%	104%	10.9	102%
413	< 60	7.4%	105%	10.6	102%
934	60 - 69	7.2%	85%	10.3	92%
1,346	70 - 79	8.3%	101%	10.6	104%
839	80 +	10.1%	112%	12.2	104%

Gender

**Figure 10:**
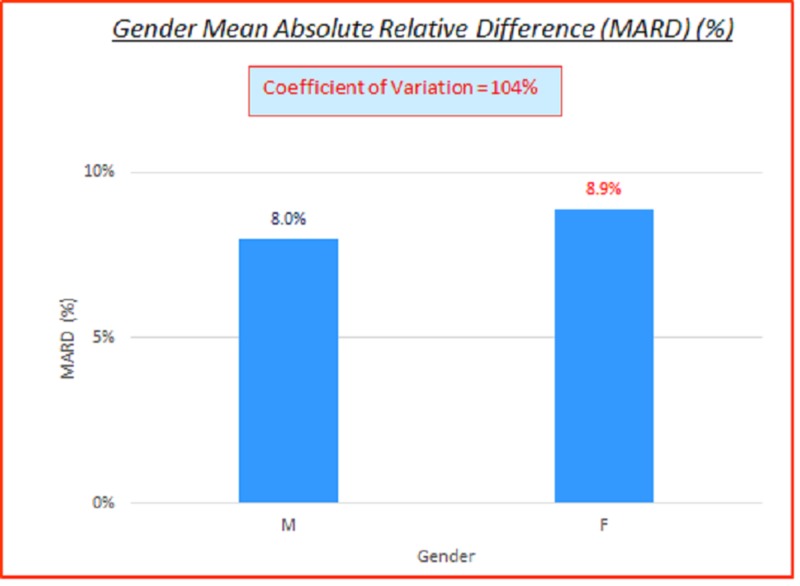
Gender MARD (%) MARD: mean absolute relative difference

**Figure 11:**
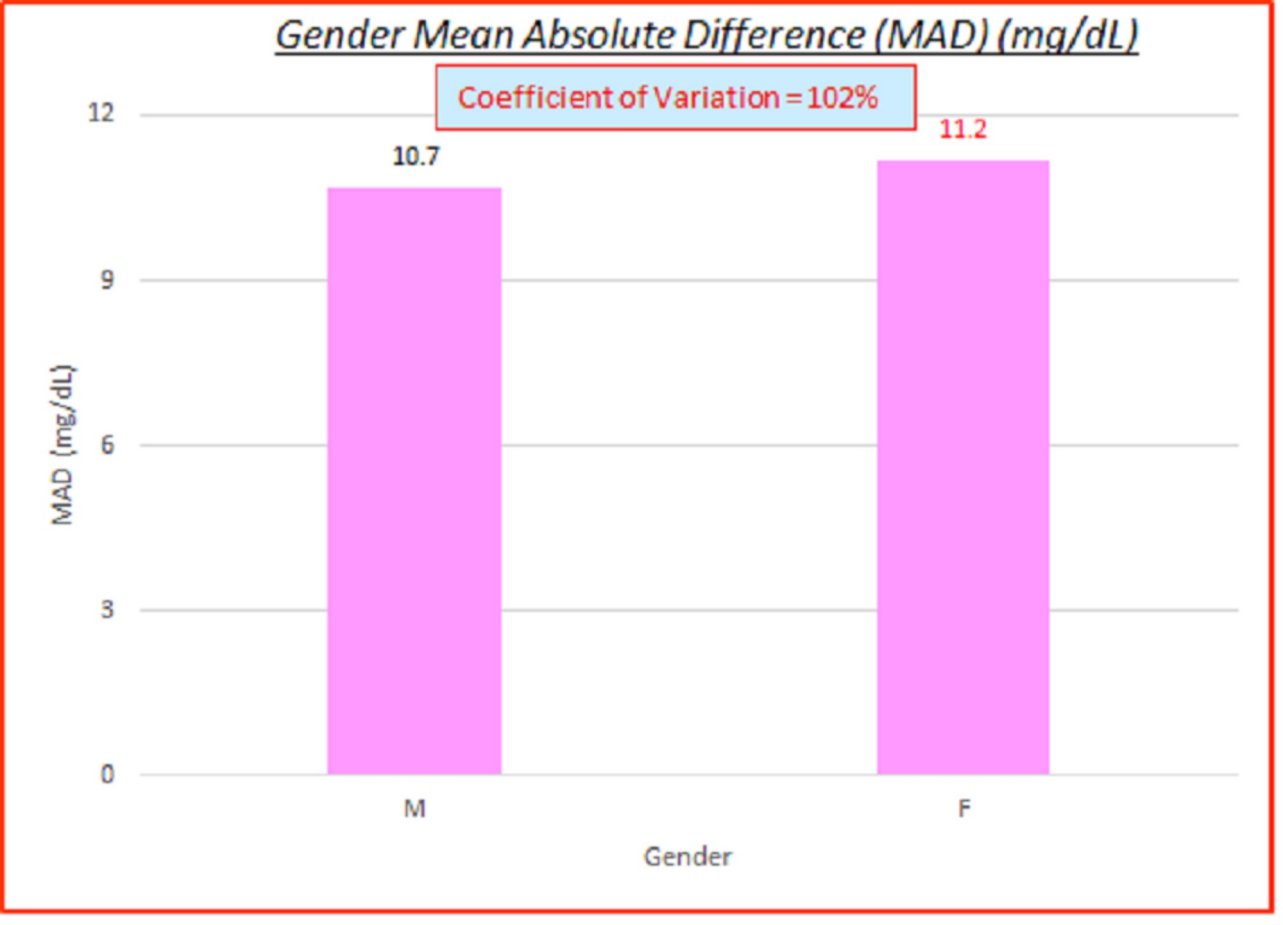
Gender MAD (mg/dL) MAD: mean absolute difference

Data parameterized according to gender are fairly consistent. ARD (%) and absolute difference (AD or GAP) (mg/dL) coefficients of variation (%) of 100% indicate high variance between the capillary BG and venous true BG data. They show a slight skewing of MARD and MAD to the female gender side. These are displayed in the data table and charts shown below in Table 6 and Figures 10-11.

**Table 6:** Gender absolute relative difference (ARD) (%) and absolute difference (AD or GAP) (mg/dL) coefficients of variation (%) MARD: absolute relative difference; AD: absolute difference; MAD denotes mean absolute difference, CV: coefficient of variation

# Data pts	Gender	MARD (%)	ARD CV (%)	MAD (mg/dL)	AD CV (%)
3,532	All	8.3%	104%	10.9	102%
2,360	M	8.0%	103%	10.7	102%
1,172	F	8.9%	105%	11.2	102%

## Discussion

SMBG devices under-estimated true BG 39% of the time, and over-estimated true BG 57% of the time. Overall MARD was 8.3%, and overall MAD was 10.9 mg/dL. Coefficients of variation of 100% or over indicate high variance between the capillary BG and true BG data. There wasn't any significant difference between the SMBG devices. There was a slight skewing of MARD and MAD to the lower BMI side, the higher age side, and the female side.

SMBG devices have been on the market for over 30 years, and they have been improved over time. Although they were designed for patients to use at home, they are also used frequently in hospitals, emergency rooms, patients' wards, physicians' offices, and senior homes for quick and convenient checking of blood glucose level.

One of the most critical uses and applications is to evaluate coma patients. Therefore, healthcare providers should be duly apprised and aware that there is a certain GAP between SMBG device measurements and true BG in order to avoid inappropriate and erroneous administration of dextrose or insulin.

There are several studies comparing SMBG device measurement and venous blood laboratory data [5-10]. Boyd et al. compared capillary and venous blood using glucometer and laboratory analyzer, and the mean difference was 0.58 mmol/L (10.4mg/dL) [5]. Sato et al. also compared SMBG devices and venous blood laboratory tests, and revealed that the mean absolute difference was 10.2 mg/dL, and MARD was 7.2% [6]. The MARDs and MADs of our extensive research are comparable to their data, but our research has far more data points over far longer follow-up periods of time.

The International Organization for Standardization (ISO) recommends that total analytical error for a glucometer be within ±15 mg/dL when laboratory glucose values are <100 mg/dL; the acceptable error should be within 15% for laboratory values ≧100 mg/dL [11]. Thus, it is not at all unexpected nor at all surprising that the overall reported MARDs and MADs were consistent with this because the SMBG devices which were used in the previous researches as well as in our study have already met the criteria before being allowed on the market. However, the extensive database underlying our research revealed large erroneous variations among the data. Absolute Differences were observed over 50mg/dL which are clearly not acceptable for medical practice.

There were 41 data points with gaps of and over 50 mg/dL. The largest gap was a triple-digit 118 mg/dL with a capillary BG of 422 mg/dL and true BG of 304. If this was in a hospital emergency room, the patient would have been administered inappropriately excessive amounts of insulin. Another case data point showed a capillary BG of 37 mg/dL and true BG for a gap of 116 mg/dL, and this patient was immediately given dextrose due to the low BG reading of the SMBG device which turned out to be incorrect as shown by the laboratory true BG data several days later. This huge gap caused inappropriate medication which could have been dangerous.

Ginsberg BH wrote in his review that glucose monitoring has some limitations regarding accuracy due to strip factors, environment factors, patient factors, and exogenous interfering substances [12]. The Committee on Standardization of Laboratory Testing Related to Diabetes Mellitus reported that there are significant device-to-device variations, and that while the devices available in Japan met the ISO/TC 212 requirements, adjustments or conversions were added to display data [13]. The committee declares that it is generally known that there is a difference between finger-pricked capillary blood glucose level and venous blood glucose level. Thus, there are many factors causing the gap between capillary BG and true BG.

All healthcare providers should therefore be keenly aware that there are certain gaps between SMBG device measurement and true BG, which could be dangerous. Although SMBG devices are convenient, caution must be duly exercised in accepting the results in medical practice, and particularly to senior female patients with low BMI.

## Conclusions

There is thus shown certain gaps between SMBG device measurements and true BG. Even though MARDs and/or MADs may meet certain criterions of accuracy, the devices themselves do indeed have certain inherent significant errors which can cause wrong medication and/or treatment that could be dangerous, particularly in the use of SMBG devices in the home where most testing is performed.
